# Supplementary material for: Chitosan-Coated Liposomes for Intranasal Delivery of Ghrelin: Enhancing Bioavailability to the Central Nervous System
Source: Pharmaceutics. 2025 Nov 19;17(11):1493. doi: 10.3390/pharmaceutics17111493 (PMC12655848; doi:10.3390/pharmaceutics17111493)
Supplement: Supplementary file 1 [file pharmaceutics-17-01493-s001.zip › pharmaceutics-3925954-supplementary.pdf]

Supplementary Material

S1. Factorial Design for Chitosan-Coated Liposomes

S1.1. Experimental Design Parameters

A factorial design was implemented to assess the effects of chitosan concentration and coating time on the main physicochemical attributes of liposomes, namely hydrodynamic diameter, polydispersity index, and zeta potential. Two independent variables were selected: chitosan concentration (% w/w) at three levels (0.1, 0.3, and 0.5) and coating time (hours) at three levels (2, 4, and 8). The response variables were analyzed to minimize particle size and polydispersity while maximizing surface charge.

S1.2. Experimental Matrix and Results

The experimental matrix and the results obtained for hydrodynamic diameter, polydispersity index, and zeta potential are summarized in Table S1.

Table S1. Experimental matrix and physicochemical responses of liposomes coated with different chitosan concentrations and coating times.

Experiment	Chitosan Concentration (% w/w)	Coating Time (h)	Hydrodynamic Diameter (nm)	Polydispersity Index	Zeta Potential (mV)
1	0.1	2	138.72 ± 4.94	0.221 ± 0.019	+20.5 ± 4.9
2	0.1	4	142 .54 ± 5.30	0.209± 0.016	+30.9 ± 3.8
3	0.1	8	145.25 ± 1.77	0.185 ± 0.035	+35.7 ± 4.9
4	0.3	2	148.40 ± 1.64	0.174 ± 0.014	+50.2 ± 5.6
5	0.3	4	152.41 ± 0.21	0.159 ± 0.018	+60.8 ± 6.6
6	0.3	8	160.76 ± 0.29	0.155 ± 0.021	+61.1 ± 4.8
7	0.5	2	210.95 ± 2.84	0.162 ± 0.01	+61.3 ± 2.9
8	0.5	4	260.84 ± 3.69	0.153 ± 0.18	+62.4 ± 3.7
9	0.5	8	294.31 ± 2.98	0.158 ± 0.019	+65.5 ± 7.9

The factorial design and the resulting data provide a comprehensive basis for optimizing chitosan-coated liposomes. This dataset supports the interpretation of the zeta potential and the relationship between formulation parameters and the physicochemical properties of the vesicles.
